# COVID-19 related multisystem inflammatory syndrome in children (MIS-C): a case series from a tertiary care pediatric hospital in Qatar

**DOI:** 10.1186/s12887-021-02743-8

**Published:** 2021-06-08

**Authors:** Mohammad Rubayet Hasan, Khaled Al Zubaidi, Karim Diab, Yahia Hejazi, Sharon Bout-Tabaku, Buthaina Al-Adba, Eman Al Maslamani, Mohammad Janahi, Diane Roscoe, Andres Perez Lopez, Patrick Tang

**Affiliations:** 1 Sidra Medicine, PO BOX 26999, Doha, Qatar; 2 Weill Cornell Medical College in Qatar, Doha, Qatar

**Keywords:** COVID-19, SARS-CoV-2, Multisystem inflammatory syndrome (MIS-C), Kawasaki disease

## Abstract

**Background:**

Multisystem Inflammatory Syndrome in Children (MIS-C) is a severe complication of coronavirus disease 2019 (COVID-19) in children, which is increasingly being reported worldwide. Here we report the first case series of 7 children diagnosed with MIS-C in Qatar.

**Methods:**

Clinical features and outcomes of COVID-19 positive patients admitted to Sidra Medicine, Qatar from June to October 2020, who met the WHO case definition for MIS-C were reviewed.

**Results:**

The mean age in our case series was 5.6 years, of which 71.4% were males. All patients were previously healthy but had a history of COVID-19 infection. Fever, rash, vomiting and abdominal pain were the most common symptoms (70–100%). The average hospitalization was 12.9 days with no case fatalities. Laboratory findings included lymphopenia and thrombocytopenia in most patients, as well as evidence of coagulopathy and elevated inflammatory markers such as C-reactive protein, ferritin and procalcitonin. Many patients (71.4%) required inotropic support in intensive care, while only one required respiratory support. Although all patients had elevated cardiac biomarkers, cardiovascular involvement was observed in 42.9% of patients with one patient developing a giant coronary aneurysm. All patients received intravenous immunoglobulin (IVIG) and 86% of patients received corticosteroids, with two patients requiring treatment with IL-1 inhibitors.

**Conclusions:**

Our report is one of the first reports on MIS-C from Asia. Although clinical features and outcomes are not significantly different from those reported elsewhere, lack of case fatalities in our cohort may indicate that early recognition and prompt medical attention is necessary for a favorable outcome in MIS-C.

**Supplementary Information:**

The online version contains supplementary material available at 10.1186/s12887-021-02743-8.

## Background

The pandemic of coronavirus disease 2019 (COVID-19) caused by severe acute respiratory syndrome coronavirus 2 (SARS-CoV-2) has had a catastrophic effect on the human population with approximately 20% of infected persons experiencing severe or critical disease, and an overall case fatality rate of 2.3% [1]. Although most children with COVID-19 have mild symptoms or have no symptoms at all, some children become severely ill needing hospitalization, intensive care, or ventilatory support. Multisystem Inflammatory Syndrome in Children (MIS-C) is a rare but serious medical condition associated with COVID-19 [2]. MIS-C is defined by inflammation in different organs such as the heart, kidneys, lungs, brain, skin, eyes, or gastrointestinal system. The causes of MIS-C remain unknown but it has been associated with SARS-CoV-2 infection [3]. Approximately 40–50% of children with MIS-C meet criteria for complete or incomplete Kawasaki disease (KD). The clinical presentation of MIS-C may also resemble that of toxic shock syndrome (TSS), secondary hemophagocytic lymphohistiocytosis, or macrophage activation syndrome (MAS) [4]. The true incidence of MIS-C is still uncertain but an estimated incidence of 0.6% among laboratory confirmed COVID-19 patients has been reported in New York [5].

To date, the majority of MIS-C cases have been reported from North America and European countries with very few reports from Asian countries [4–9]. Large case series conducted in the USA and UK show that risks associated with developing MIS-C may vary by gender, age and ethnicity. Although male gender and black and Hispanic races were predominantly affected [4–6], it is possible that MIS-C among Asians are under-represented because of under reporting. In this study, we aim to review and summarize the clinical presentation, laboratory parameters, outcome and management of MIS-C cases presenting to a tertiary care pediatric hospital in Qatar and compare them with previously published cases in other countries.

## Methods

Sidra Medicine is a 400-bed women’s and children’s tertiary care hospital in Qatar. MIS-C cases were identified by querying in the electronic medical record of children with COVID-19. Probable cases brought to the attention of infectious disease physicians and medical microbiologists were also included. Only patients who met the World Health Organization (WHO) case definition of MIS-C were selected for chart review. Data were recorded in a standardized form and deidentified. Descriptive statistics were performed and presented as mean and standard deviation (±/SD) for continuous variables or as number and percentages for nominal/categorical variables.

## Results

At the time of this report, there were approximately 138,000 COVID-19 cases and 237 associated deaths reported in Qatar. Since the initiation of COVID-19 screening at Sidra Medicine (April 16 to Nov. 21, 2020), a total of 28,653 COVID-19 tests were performed of which 7812 were on individuals < 18 years old. During this period, a total of 167 children were positive for COVID-19 by RT-qPCR, and 7 of these patients fulfilled the WHO criteria for MIS-C and were managed in our hospital. The mean age at diagnosis was 5.6 ± 2.7, and the majority of the cases were male (71.4%) (Table 1). All patients were previously healthy. Five out of 7 cases were initially admitted to the pediatric intensive care unit (PICU), primarily for vascular support. All patients were managed according to the diagnostic and treatment algorithms established in May 2020 by a multidisciplinary group of pediatricians and subspecialists in Qatar, in response to COVID-19 pandemic and the emergence of MIS-C cases in Europe and the United States.

**Table 1:** Patient characteristics and clinical presentation

	Case-1	Case-2	Case-3	Case-4	Case-5	Case-6	Case-7	Summary
***Demographics***								
Age	6	6	3	7	7	9	1	Mean, 5.6 ± 2.7
Gender	Male	Male	Male	Male	Female	Female	Male	Male, 71.4%
***Clinical presentation***								
Fever	Yes	Yes	Yes	Yes	Yes	Yes	Yes	100%
Rash	No	Yes	Yes	Yes	Yes	Yes	Yes	85.7%
Tachycardia	Yes	No	Yes	Yes	No	Yes	Yes	71.4%
Tachypnea	No	Yes	No	No	Yes	No	No	28.6%
Hypotension	Yes	Yes	No	Yes	No	No	No	42.9%
Abdominal pain	Yes	No	Yes	^a^Yes	Yes	Yes	No	71.4%
Diarrhea	No	No	Yes	No	No	Yes	Yes	42.9%
Vomiting	Yes	Yes	Yes	Yes	Yes	Yes	Yes	100%
Decreased oral intake	Yes	Yes	Yes	No	No	No	Yes	57.1%
Cough	Yes	No	No	No	No	No	No	14.3%
Sore throat	No	Yes	No	No	No	No	No	14.3%
Conjunctivitis	No	Yes	No	Yes	No	Yes	Yes	57.1%

^a^Patient underwent laparoscopic appendectomy

Fever and rash were the most common presenting symptoms among the MIS-C cases in our hospital with 100 and 85.7% of the patients experiencing these symptoms, respectively (Table 1). Additionally, gastrointestinal symptoms were common among these patients with 100, 71.4 and 42.9% patients presenting with vomiting, abdominal pain and loose stools, respectively. Upper respiratory tract infection (URTI) symptoms were less prevalent in our study group, with cough and sore throat experienced by one patient each, and conjunctivitis in 3 other patients. Two of the cases were suspected to have urinary tract infection (UTI) based on initial urine microscopy, however none had urinary tract symptoms at the time of presentation or had a positive urine culture after presentation.

Two of the cases had previous positive RT-qPCR results for SARS-CoV-2 (Table 2). At presentation, only one case had positive nasopharyngeal swab (NPS) for SARS-CoV-2. Two of the remaining 4 cases were initially negative by RT-qPCR in NPS but were later found to have positive COVID-19 serology. Additional RT-qPCR testing for these patients using nasopharyngeal wash (NPW) specimens confirmed the presence of SARS-CoV-2 RNA. Of the 6 children who were tested for antibodies to SARS-CoV-2, all were positive.

**Table 2:** Laboratory results

	Case-1	Case-2	Case-3	Case-4	Case-5	Case-6	Case-7	Summary
***COVID-19***								
RT-qPCR	NPS-Neg NPW-Pos	NPS-Neg NPW-Pos	NPS-Pos	NPS-Neg	^a^NPS-Pos	^a^NPS-Pos	NPS-Neg	71.4% cases positive in at least one specimen
Serology	Positive	Positive	Positive	Positive	Not done	Positive	Positive	6/6, 100% positive
***Hematology***								
WBC (10^9^/L)	27.3	19.4	16	9.7	16.9	6.9	24.1	71.4% above range (Ref: 4–14)
Neutrophil (10^9^/L)	24.4	16.5	5.3	9.5	13.9	4.6	16.1	71.4% above range (Ref: 0.8–7.2)
Lymphocyte (10^9^/L)	0.9	0.8	1.2	0.2	2.1	0.6	6.5	71.4% below range (Ref: 1.3–8)
Platelets (10^3^/mL)	140	80	570	105	116	105	900	71.4% below range (Ref: 150–400)
***Inflammatory markers***								
CRP (mg/L)	262.2	228.3	162	304.5	93	82.8	143	100% above range (Ref: 0–7.5)
Ferritin (ng/mL)	324	581	377	334	326	341	621	100% above range (Ref: 10–56)
PCT (ng/mL)	21.6	7.22	9.4	> 50	2.15	Not done	0.59	6/6, 100% above range (Ref: < 0.1)
IL-6 (pg/mL)	35	4	Not done	Not done	2665	Not done	100	3/4 above range (Ref: 0–16.4)
***Coagulation***								
PT (sec)	16.8	15.1	18.3	17	17.5	15.9	12	83% above range (Ref: 11.7–15.1)
D-dimer (mg/L)	7440	2266	7500	> 7500	3538	2381	3060	100% above range (Ref: ≤500)
Fibrinogen (mg/dL)	4	3.9	3.4	4.4	3.7	3.6	4.3	28.6% above range (Ref: 1.6–4)
***Cardiac***								
Troponin (ng/L)	40	14	68	309	161	34	4	100% above range (Ref: 0–0.4)
NT-proBNP (ng/L)	5253	7006	2314	2874	592	506	1444	100% above range (Ref: < 125)

*NPS* nasopharyngeal swab; *NPW* nasopharyngeal wash; *WBC* white blood cell; *CRP* C-reactive protein; *PCT* procalcitonin; *PT* prothrombin time; *NT-proBNP* N-terminal B-type natriuretic peptide

^a^Previous positive

All patients had extensive laboratory workup done upon admission or at the time when MISC was suspected (Table 2). Although total white blood cell (WBC) counts were variable among our study population with a range between 6.9 to 27.3 (10^9^/L), 5 of 7 cases were lymphopenic for their age. Additionally, 5 cases had a low platelet count for their age, although none had severe thrombocytopenia. All of our MIS-C cases showed a hyperinflammatory status with remarkably high C-reactive protein (CRP), procalcitonin (PCT) and ferritin levels, and deranged coagulation profile. IL-6 was high in 3 of 4 cases who were tested during their hospital stay.

Chest radiography was performed on 6 of 7 patients (Table 3). The most commonly described abnormalities were bilateral perihilar infiltrates and peribronchial thickening. Bilateral interstitial opacities and pulmonary edema were described in just one patient. Abdominal ultrasound (US) was performed on 6 of 7 patients. The most significant finding was that of an aortic aneurysm in one patient. The remaining patients had a variety of non-specific findings including increased echogenicity of the liver, gall bladder wall edema and thickening, bulky and echogenic kidneys, enlarged mesenteric lymph nodes, pleural effusions and ascites.

**Table 3:** Clinical outcome

	Case-1	Case-2	Case-3	Case-4	Case-5	Case-6	Case-7	Summary
Hospital length of stay (days)	12	10	6	20	7	8	27	Mean, 12.9 ± 7.8
ICU stay (days)	12	10	None	11	4	3	None	71.4%
Shock	Yes	Yes	None	Yes	None	Yes	None	57.1%
Abnormal echocardiogram	Yes	No	No	No	Yes	No	Yes	42.9%
Abnormal EKG	Low voltage in limb leads	Not done	Not done	Initial ECG RBBB	No	No	Deep Q wave in inferior leads	42.9%
LAD/RCA z-score ≥ 2.5	No	No	No	No	No	No	^a^Yes	14.3%
Pericardial Effusion	Minimal	No	No	No	No	No	No	14.3%
Ejection Fraction	51%	65%	68%	65%	54%	69%	70%	28.6% below range (Ref: < 55%)
Mitral valve regurgitation	Mild	No	Trivial	No	Mild	No	No	42.9%
Abnormal CXR	Yes	Yes	Yes	Yes	Yes	Not done	Yes	6/6, 100% abnormal
Pleural effusion	Small bilateral	No	No	No	Small right sided	No	No	28.6%
Mechanical ventilation	None	None	None	Yes	None	None	None	14.3%
Abnormal US abdomen	Yes	Yes	Yes	No	Yes	Not done	Yes	5/6, 83.3% abnormal

*CXR* Chest X-ray; *US* ultrasound

^a^LAD large aneurysm 9.5 mm (Z score + 31.44), RCA small aneurysm 3.1 mm (Z score + 4.16), LMCA medium aneurysm 5.2 mm Z score + 7.75

**Fig. 1:**
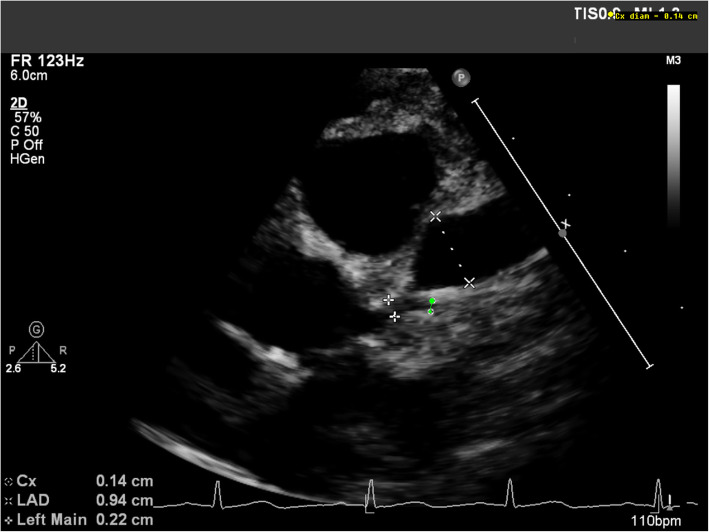
Coronary artery aneurysm in a MIS-C patient (Case 7). Echocardiographic short axis view of the left coronary artery system showing the mildly dilated main left coronary artery and the giant aneurysm in the left anterior descending coronary artery with the respective measurements (Video in the supplemental file). LMCA: Left Main Coronary Artery; Cx: circumflex; LAD: Left Anterior Descending


Echocardiograms were performed on all patients at diagnosis with at least 4 weeks of follow-up, and after 8 weeks or earlier for patients with abnormal findings (Table 3). Cardiovascular involvement was seen in 3 of 7 patients in our study group (42.9%). Two patients had transient ventricular dysfunction with ejection fraction (EF) < 55%. Five patients (71.4%) received vasoactive support. All patients had elevated levels of N-terminal B-type natriuretic peptide (NT-proBNP) and troponin (Table 2). None of our cases had arrhythmias even in the acute stage. Coronary-artery aneurysm was identified on the basis of a z score of 2.5 or higher in the left anterior descending (LAD) or right coronary artery (RCA) in one patient (Table 3; Case-7) who developed a giant aneurysm in the left anterior descending (LAD) coronary artery (initially 4.9 mm, z-score > 10). This patient also had a dilated left main coronary artery measuring 3.9 mm (z-score + 3.7) and a dilated right coronary artery measuring 2.6 mm (z-score + 2.7) (Fig. 1, Supplemental video). The patient was placed on anticoagulation and dual antiplatelet therapy in addition to two doses of intravenous immunoglobulin (IVIG) and interleukin-1 (IL-1) inhibitor (anakinra). His LAD aneurysm enlarged to 9.5 mm (z-score + 31.4) and was still present on the latest follow-up after 8 weeks from diagnosis.

**Additional file 1.** Coronary artery aneurysm in a MIS-C patient. Video of echocardiogram performed on a MIS-C patient with coronary artery aneurysm.

The mean hospital stay of our MIS-C patients was 12.9 days, with 5 initially requiring intensive care management for ionotropic support (Table 3). Only one case (Case-4; Table 3) presented with acute respiratory distress syndrome (ARDS) and required mechanical ventilation. This patient also had prolonged fever and required 2 doses of IVIG, pulse steroids, and anakinra after no response to the initial measures. Broad spectrum antibiotics were initiated in all of the cases after consultation with the infectious disease team (Table 4). Patient 1 initially received cefepime and vancomycin for suspected urosepsis, but his treatment was later upgraded to meropenem and vancomycin due to a lack of response. Antibiotic treatment was de-escalated after all culture results were negative and the patient was tested positive for COVID-19. Patient 3 received ceftriaxone and metronidazole as post-appendectomy prophylactic treatment. All other patients received antibiotics for suspected infection while waiting for culture results. Aspirin was given to all patients during their hospital stay and on discharge for coronary thrombosis prophylaxis. All of our patients recovered and were discharged from the hospital in good clinical condition.

**Table 4:** Treatment

	Case-1	Case-2	Case-3	Case-4	Case-5	Case-6	Case-7	Summary
IVIG	2 g/kg; 1 dose	1 g/kg; 1 dose	2 g/kg; 2 dose	1.5 g/kg; 1 dose	2 g/kg; 1 dose	2 g/kg; 2 dose	2 g/kg; 2 dose	100%
^a^Corticosteroids	Prednisolone (2 mg/kg/D;1 M)	Prednisolone (2 mg/kg/D; 2 W)	None	Methylprednisolone (30 mg/kg/D; 3D) Prednisolone (1 mg/kg/day; 1 M)	Methylprednisolone (30 mg/kg/D; 1 dose) Prednisolone 2 mg/kg/day; 6 W)	Methylprednisolone (30 mg/kg/D; 3D) Prednisolone 2 mg/kg/day; 3 M)	Prednisolone (2 mg/kg/D; 3 M)	85.7%
Antibiotics	FEP (2D) MEM (5D) VAN(3D) AMC (7D)	CRO (7D) CLI (7D)	CRO (5D)	CRO (3D) MTZ (4D)	MEM (1D) TZP (5D)	CRO (2D)	None	100%
Anticoagulants	Enoxaparin (2 mg/kg/D; 3D)	Enoxaparin (2 mg/kg/D; 15D)	None	Enoxaparin (2 mg/kg/D; 15D)	Enoxaparin (1 mg/kg/D; 4D)	Enoxaparin (1 mg/kg; 2 dose)	Enoxaparin (2 mg/kg/D; 6 M) Clopidogrel (10 mg/D; 6 M) Warfarin (2 mg/cont.)	85.7%
Epinephrine/ norepinephrine	EPI (0.1 μg/kg/min, 2D) N-EPI (0.1 μg/kg/min, 4D)	N-EPI (0.15 μg/kg/min, 2D)	None	N-EPI (0.05 μg/kg/min, 4D)	None	N-EPI (0.1 μg/kg/min, 1D)	None	42.9%
Aspirin	4 mg/kg/D; 1 M	5 mg/kg/D; 1 M	5 mg/kg/D; 3 M	Yes	2 mg/kg/D; continued	3 mg/kg/D; 3 M	5 mg/kg/D; continued	100%
^a^Interleukin-1ra inhibitor	None	None	None	Anakinra (5 mg/kg/D; 2 M)	None	None	Anakinra (4 mg/kg/D, 2 M)	28.6%

^a^duration includes tapering

*M* month; *W* week; *D* day; *IVIG* intravenous immunoglobulin; *AMC* amoxicillin-clavulanic acid; *MEM* meropenem; *FEP* cefepime; *CRO* ceftriaxone; *CLI* clindamycin; *TZP* piperacillin-tazobactam; *EPI* epinephrine; *N-EPI* norepinephrine

## Discussion

This case series describes 7 cases of MIS-C in our hospital. Similar to earlier reports, all patients were previously healthy and presented at our hospital approximately 4–6 weeks after the peak of the COVID-19 outbreak in the country [4]. In most cases, MIS-C was suspected early because of a history of COVID-19 infection based on RT-qPCR or serology. In two cases who were initially negative by PCR, antibody testing was useful to determine the COVID-19 infection status of the suspected MIS-C patients. Overall, 71.4% of our patients had positive COVID-19 PCR results as compared to ~ 50% of positive COVID-19 PCR results reported in other studies [10, 11]. The fact that all patients in our case series who were tested for SARS-CoV-2 antibody were positive at the time of presentation supports the post-infectious nature of the disease [12].

The clinical presentations of MIS-C patients in this case series were mostly similar to earlier reports with fever and gastrointestinal problems being the most common initial symptoms. [11, 13] In our experience, abdominal pain in these patients was severe in nature and resembled appendicitis. In fact, one of our patients underwent appendectomy, which subsequently showed a normal appendix. Respiratory symptoms were less prominent in our cohort with only one case having cough and another being intubated as part of ionotropic support without significant lung pathology. This is consistent with previous studies although some studies have reported a higher percentage of cases requiring respiratory support during their illness [10, 13, 14]. The signs and symptoms of patients in our case series differed from a recently published study on Latin American children which showed higher rates of upper and lower respiratory tract infections and a lower percentage of gastrointestinal symptoms among the MIS-C patients [15].

Significant cardiac involvement in cases of MIS-C has been documented in recent reports highlighting the common similarity with KD. This emphasizes the need for cardiac evaluation with echocardiography at diagnosis and at regular intervals consistent with the management of KD [4, 16]. It is reported that up to 56% of cases can have decreased systolic ventricular function with EF < 55%, the most common cardiac abnormality seen in these patients [13, 16], which in contrast to KD has less propensity for significant ventricular dysfunction [14]. In our small case series, although approximately three quarter of patients required vasopressor support, only two had transient LV dysfunction that recovered within a short period, which is in contrast with patients with KD who rarely present with hemodynamic instability [4].

Elevated troponin levels have been previously associated with poor outcome in patients with COVID-19 and could be a reflection of the degree of systemic inflammation and myocardial effects [17]. Elevated troponin, NT-proBNP and D-dimer levels were also commonly noted in our case series (Table 2). Of particular interest is the degree of coronary artery involvement in MIS-C cases which occurred in one case with the patient developing a giant aneurysm in the LAD and dilatation of both the left main and right coronary arteries. Coronary artery involvement in MIS-C cases is reported to occur in up to 15% of cases in a recent report with few patients developing giant aneurysms. [11, 14, 16, 18] Although the true incidence of such involvement is still to be defined, it seems similar to that in KD where it occurs in about 25 and 4% of untreated and treated patients, respectively [19]. In addition, no clear predisposing factors were identified for those with higher risk for development of coronary involvement in MIS-C cases.

Although the pathophysiology of MIS-C is poorly understood, it has been suggested that it is a post-infectious process triggered by an abnormal immunophenotype that is distinct from KD, MAS, and cytokine release syndrome [2]. Elevated inflammatory markers and evidence of coagulopathy are common laboratory findings and are among the most important criteria for the clinical diagnosis of MIS-C. However, the correlation between the two in MIS-C patients is not known. It has been suggested that ‘cytokine storm’ or the enhanced production of inflammatory cytokines, especially IL-6, may lead to the activation of the coagulation cascade in COVID-19 patients [20, 21]. In our study, coagulation dysfunction in MIS-C patients was seen in terms of markedly elevated D-dimer and abnormal PT. However, fibrinogen levels were slightly elevated in only 2 patients and IL-6 levels were not available for all patients. Therefore, the role of IL-6 or any other inflammatory cytokines remained inconclusive in our study, but notably, one patient (case 5) who had exceptionally high level of IL-6 did not have proportionally higher levels of coagulation markers. This is in contrast to the findings of a study which showed a positive correlation between IL-6 level and fibrinogen level in patients with COVID-19 associated ARDS [22]. Other studies suggest that the cytokine storm in MIS-C patients differs from those in severe acute COVID-19 and hypothesize the role of autoantibodies such as lupus anticoagulants in COVID-19 associated coagulopathy and thrombosis [23–25].

Previous studies showed a death rate of 1.7–1.8% [13, 26]. Fortunately, all of the cases in our case series had a favorable outcome, with no deaths. All patients received aspirin for coronary thrombosis prophylaxis and one patient with giant aneurysm was placed on dual antiplatelet therapy according to American Heart Association (AHA) guidelines for treating patients with Kawasaki disease [19]. It was also noteworthy that all 7 patients except one were initially treated with antibiotics. Although there is a great concern about the overuse of antibiotics in children with COVID-19 [27], it should be pointed out that the substantial overlap between the clinical presentation of MIS-C and other life-threatening bacterial infections such as sepsis and toxic shock syndrome, likely justify the empirical use of broad-spectrum antibiotics in these patients until a diagnosis is established and negative culture results are available [28]. Biomarkers that accurately rule out bacterial infections in patients with an equivocal clinical presentation could reduce antibiotic exposure in patients with MIS-C.

With the exception of two cases, all patients in our case series initially required PICU care with inotropic support being the main reason for PICU admission, consistent with previous reports of MIS-C [4, 10, 13, 29]. The majority of our cases responded well to IVIG with or without intravenous (IV) corticosteroids in terms of subsidence of fever and decreased need for inotropic support. Only two cases required two doses of IVIG and IL-1 inhibitor, as these patients had a more complicated course with ARDS and coronary aneurysm, respectively. These patients also had prolonged fever, which did not respond to initial measures. A similar pattern in clinical response was noted in less than 10% of cases requiring IL-1 or IL-6 antagonists in a recent systematic review [13]. Due to the similarities between MIS-C and KD and their cardiac involvement, current treatment strategies are similar from cardiac point of view [4, 13]. However, the long-term outcomes of MIS-C, such as the sequelae of coronary artery aneurysm formation, remain unknown. The benefit of longer-term cardiac follow up to evaluate the effects on cardiac function and persistence or regression of coronary aneurysms remain to be determined.

## Conclusions

We report the first case series of COVID-19 associated MIS-C in Qatar. Our patients commonly presented with fever, rash and gastrointestinal symptoms and required intensive care. Most common laboratory findings include lymphopenia and thrombocytopenia and elevated CRP, ferritin, PCT, D-dimers, PT, NT-proBNP and troponin. Only one patient had acute respiratory distress syndrome (ARDS) and required respiratory support, and cardiovascular involvement was observed in approximately 43% of patients, with one patient with coronary-artery aneurysms. All patients were treated with IVIG, and some received corticosteroids and IL-1 inhibitors; all patients were fully recovered.

## Data Availability

All data generated or analysed during this study are included in this published article [and its supplementary information files].

## Abbreviations

COVID-19: Coronavirus disease 2019
MIS-C: Multisystem inflammatory syndrome in children
WHO: World Health Organization
IL-1: Interleukin - 1
SARS-CoV-2: Severe acute respiratory syndrome coronavirus 2
KD: Kawasaki disease
RT-qPCR: Reverse transcriptase – quantitative real time polymerase chain reaction
PICU: Pediatric intensive care unit
URTI: Upper respiratory tract infection
UTI: Urinary tract infection
NPW: Nasopharyngeal wash
WBC: White blood cell
CRP: C-reactive protein
PCT: Procalcitonin
IL-6: Interleukin - 6
US: Ultrasound
EF: Ejection fraction
NT-proBNP: N-terminal B-type natriuretic peptide
LAD: Left anterior descending
RCA: Right coronary artery
ARDS: Acute respiratory distress syndrome
IVIG: Intravenous immunoglobulin
IV: Intravenous
